# Valuing Simplicity: Developing a Good Point of Care Diagnostic

**DOI:** 10.3389/fsoc.2020.00037

**Published:** 2020-05-22

**Authors:** Nora Engel, Anja Krumeich

**Affiliations:** Department of Health, Ethics & Society, Care and Public Health Research Institute, Maastricht University, Maastricht, Netherlands

**Keywords:** simplicity, valuing, point-of-care diagnostics, tuberculosis, HIV

## Abstract

Point-of-care testing promises laboratory-based precision in settings that do not have easy access to laboratories or where processing tests takes too long or is too costly. Developers and global health actors stress values and norms such as simplicity, rapidity and accuracy for realizing diagnostic innovations that work at point of care and are aligned to the specific requirements of point-of-care settings. This paper uses fieldwork among diagnostic manufacturers, scientists, donors, members of civil society, industry consultants, international organizations, regulators, policymakers, programme officers, lab technicians, and clinicians involved in development and implementation of tuberculosis and HIV diagnostics, to examine how norms and values of what constitutes a good point-of-care diagnostic are operating in practice through both top-down and bottom-up dynamics. It draws the link between design, evidence and adoption of diagnostics and how the different actors interpret the values underpinning the new practice. The analysis draws on literature on valuation practices, evidence-making and technology design in science and technology studies and medical sociology. The findings reveal how these values constitute innovation, implementation, and evaluation practices across global and local (India) sites, with important consequences for funding of health infrastructure, capacity, and training resources for addressing some of the existing structural inequalities. Dominant values currently defining point of care diagnostics risk exacerbating health inequalities between those who do and do not have the necessary social and financial resources to access better equipped and functioning healthcare facilities, especially in resource constrained settings.

## Introduction

What is a good point-of-care (POC) diagnostic? The idea of diagnosing at point of care (i.e., where patients encounter the healthcare system, for instance in communities and clinics) has gained much enthusiasm and attention among researchers, policymakers and funders in the last decades. A diagnosis transforms illness into actual disease. As the classification tool of medicine, diagnosing is an act that marks a boundary between the normal and the pathological (Jutel, 2009; Jutel and Nettleton, 2011). By allowing healthcare workers to diagnose without relying on laboratories and laboratory technicians, POC diagnostics change when and where the boundary between the normal and the pathological is drawn and who has the power to make these decisions. A counselor using an HIV rapid test in a remote clinic, for instance, is able to make a treatment decision based on that test result without involvement of the laboratory technician in a faraway centralized laboratory or a clinician who might not be present every day. The promise is that such diagnostics can improve management of infectious diseases by cutting treatment delays that are often associated with conventional laboratory-based testing, avoiding increased morbidity or drug resistance and improving access to quality care in settings where laboratory-based testing takes too long, is too costly or unavailable (Peeling and Mabey, 2010; Pant Pai et al., 2012). The understanding of how such POC technologies should look like has evolved over time. Initial ideas of a simple dipstick test that can be done without equipment (like a pregnancy test) are evident in the World Health Organization's (WHO) ASSURED criteria: affordable, sensitive, specific, user friendly, rapid and robust, equipment-free, and delivered (Peeling and Mabey, 2010). More nuanced understandings emphasize the rapid completion of test and treat cycle within one patient encounter (“while the patient waits”). This latter perspective acknowledges that in order to complete processes of diagnosing at POC successfully, POC diagnostics will occupy a spectrum of technologies (from dipsticks to automated molecular tests, portable analysers, and imaging systems), users (lay persons to highly trained), and settings (homes, communities, clinics, to peripheral labs and hospital wards) (Pant Pai et al., 2012).

In this paper, we are interested in understanding the norms and values of a good POC diagnostic that different actors involved in diagnostic development and implementation enact and through what practices they do so. How do norms and values of what constitutes a good POC diagnostic operate in practice? Through fieldwork and interviews with global diagnostic developers, donors, members of civil society, industry consultants, international organizations, regulators, and researchers involved in innovating POC diagnostics for tuberculosis (TB) and HIV, as well as local implementers, developers, and users in India, we found that simplicity remains a prominent value also when developing more sophisticated technologies for POC. Simplicity is assumed to guarantee accessibility. Yet, we know from earlier research that in practice this is not always the case. In our earlier work on diagnosing at POC in India we showed that diagnosis is not always achieved, the multiplicity of diseases and diagnoses not always made manageable or coordinated even in the presence of what is believed to be simple and rapid POC diagnostics. This has to do with the backdrop of the healthcare system. The fragmented and pluralistic Indian health system requires of patients and providers to be super coordinators (Engel et al., 2017b; Yellappa et al., 2017). Yet typically, the burden of the failure is put on the difficult circumstances rather than the funders and designers of diagnostics. The complexity of a health system, the multiplicity of diseases, explanations, providers, tools and actors, contrasts with the lure of simple tools. Similarly, ethnographic case studies in Uganda, Tanzania and Sierra Leone show that rapid tests, such as those for malaria, might be simple to use in and by themselves, but making them work in a weak or economically constrained health system is not simple at all. On the contrary, it might require precisely the type of medical expertise, organizational capacity and equipment these devices were designed to replace (Beisel et al., 2016). This is counter-intuitive, because simplicity was assumed to be an enabler to make these devices work within existing weak or under-resourced systems, workflows, and constrained capacities. The lure of simplicity also leads to systematic underinvestment in training and monitoring of staff capacity resulting in incorrect performance and misinterpretation of POC diagnostics. Again, counter-intuitively, the training needs of staff at POC unfamiliar with diagnostic techniques might be even greater than for laboratory-based tests operated by laboratory personnel. Training of POC staff might need to include good laboratory practice, quality control, safety, and instrument maintenance apart from how to conduct the diagnostic (Palamountain et al., 2012).

Simplicity of global health technologies, those designed to work in (resource-constrained) situations across the Global South and North, is often associated with their mobility and portability. Medecins Sans Frontieres developed humanitarian kits to make medical equipment and guidelines travel to remote locations and resource constrained settings. These kits allow simplifying the procedures on site and are at the same time standardizing between locales (Redfield, 2013). When technologies travel to another situation, standardization, automation, and simplicity are believed to prevent too much fluidity (change, adaptation, and deformation); to make these technologies sticky and ensure sameness and accuracy across sites. Sticky technologies, such as the plumpynut, lie between Latour's immutable mobiles and de Laet and Mol's fluid technologies. They retain shape, are much firmer than a fluid technology but still mutable (Scott-Smith, 2018). Fluidity can be a recipe for success, as in the case of the Zimbabwean bush pump and its adaptable, flexible and responsive nature (de Laet and Mol, 2000). Yet, fluidity can turn out to be dangerous too, as the case of a former German ambulance converted into a Ghanaian minibus shows. In Ghana, traffic accidents involving old cars are a serious health hazard. The former ambulance car is robust and fluid enough to continue functioning as a means of public transport, but the continued use of overaged cars brings real life-threatening dangers to public transport (Beisel and Schneider, 2012). A POC diagnostic that is too fluid could be dangerous as well, because it needs to retain shape and ensure sameness and quality of results across different testing sites and bodies.

Science and technology studies scholars (STS) have highlighted that heterogeneous actors, multiple things and steps are involved and need to be coordinated when making technologies work: Seemingly simple technologies, such as the Pap smear, include more than just the testing kit, swab and brush but also all the people, bodies, things, infrastructures, places, and activities that produce a pap smear result (Clarke and Casper, 1996). This understanding of technology and practice also has implications for how sameness is produced. Even simple laboratory procedures are performed differently (Jordan and Lynch, 1992). The understanding that diagnostics produce sameness is not only dependent on a technology's black-boxed or standardized nature, but also emerges out of the assumptions and agreements among researchers' activities and practices (Fujimura, 1996). In this case, the assumptions around simplicity and what guarantees sameness or accuracy of diagnostic results at POC are important values to study.

In assigning value to diagnostics, the different actors involved make use of what emerging literature has termed “valuation frames” (Bessy and Chauvin, 2013) or registers that actors use when valuing. This literature has pointed out that the way these registers come into play depends on the particular actor's practices and on the situation, and that these registers change over time (Heuts and Mol, 2013; Dussauge et al., 2015). We will examine how during design, development, implementation and evaluation of POC diagnostics, different actors value simplicity, and how other value registers such as biosafety, cost, accuracy, or workflow interact with it. Our approach allows analyzing technology both at the level of upstream design and development and at the level of implementation. Our analysis reveals that it matters what kind of enactments of simplicity make it into practice, which value registers dominate and what that means for access and utilization.

## Valuation as Practice

STS scholars have studied valuation as a social practice, how values are enacted in actions, in technical practices and in valuation practices. These studies do not take values as a given but as things to be explored (Helgesson and Muniesa, 2013). Such a perspective asks: What are power struggles over which values become dominant? How are different valuation practices coordinated? How do valuation and values shift over time? At one point value is tied to a biomedical platform, then to a patent then to patient impact or the possibility of a medical intervention. How are different or contradicting values weighed against each other? Whose values are important when tied to people or organizations (Dussauge et al., 2015)?

In this line of scholarship, Heuts and Mol analyzed how tomato growers, sellers, developers, professional cooks and consumers value a good tomato (Heuts and Mol, 2013). The authors highlight how in their practices, actors draw on different registers for valuing tomatoes including monetary (cost), handling (such as firmness), historical time (what a good tomato is changes), naturalness (organic, chemicals), and sensual/aesthetic appeal. A professional cook cares whether a tomato is juicy enough to be used in a salad or not so juicy when preparing a sandwich. A tomato seller, on the other hand, values a tomato depending on how well it withstands transportation. Hence, these registers are not given, but embedded and enacted in the particular practices of each actor, contradicting or dominating over others, at times. The price of a tomato might trump its naturalness or tastefulness. The tomato we find in the supermarket is not necessarily the tomato the farmer produced. It is the outcome of competition between different actors' registers in the process of which taste in a consumer's register might have been overruled by other actors' valuation practices (Heuts and Mol, 2013).

To examine what makes a good POC diagnostic, we will use the idea of valuing as a practice, and different registers that are pushing/pulling and competing and how, as a result, what is considered a “good” diagnostic changes. In order to deal with the public health and global policy character of our case, we will use insights from critical policy analysis to delineate dominant value registers (and interests) to understand how some actors have more power in defining what is considered of value and for whom (Gusfield, 1981; Bacchi, 2009; Stone, 2012).

## Methods

This paper draws on ethnographic fieldwork on the development of TB and HIV diagnostics for the POC. Below we introduce some of the technical background and policy context of TB and HIV diagnostic innovation. Accordingly, we have purposefully sampled participants for our study. Taken together, examples from the two diseases allow covering different parts of the innovation and implementation process and related valuation practices.

Avoiding diagnostic delay, continuous monitoring of disease status, emergence of drug resistance as well as linkage to care are crucial concerns relevant to the control of both these diseases. Yet, the policy context of HIV and TB differs with regard to cultures of care. HIV care has been historically more attuned to patient-centered concerns, such as stigma. TB care has been approached from a traditional public health approach with a major focus on reducing transmission (i.e., by ensuring treatment adherence through direct observation). Further, HIV and TB also differ with regard to diagnostic development. In the past decades, HIV has had more attention and development efforts related to diagnostics, particularly with regard to rapid diagnosis. What is more, diagnosing HIV is technically less challenging than TB. Blood samples used to diagnose HIV are easier to work with and require less preparatory steps than sputum, the main specimen used globally in TB control. For decades, the options of diagnosing TB in clinics were limited to sputum smear microscopy, a test that is over 100 years old and often inaccurate, chest X-rays and clinical judgement. In recent years, a few new diagnostics have been developed. Most notable for the purpose of this paper is the Xpert MTB/RIF by Cepheid, a molecular test of TB and resistance to rifampicin (one of the main anti-TB drugs). The test involves a platform with, depending on the model, four cartridges connected to a computer and provides results in 90 min. It was heralded to revolutionize TB control as a new “while you wait test” to be used outside conventional laboratories, partly due to its simplicity, accuracy, and rapidity (World Health Organization, 2010). The WHO now recommends its use as the initial diagnostic test for TB, while acknowledging resource implications due to the higher cost of the assay (World Health Organization, 2016). Yet, challenges remain with access, implementation and utilization (Albert et al., 2016). Following these developments, other test developers are working toward similar molecular based TB diagnostics for the POC. The majority of these developers are based in the US/EU and a few in India and China. They are typically smaller start-ups with a technology that they are trying to adapt for TB. The field of HIV has a long history of testing at POC with disposable rapid tests kits produced by many different established diagnostic companies. Most of these kits use lateral flow technique (similar to a pregnancy test), rely on a finger-prick blood sample and take up to 20 min to perform. Instrument-based testing for HIV at POC is much less common. Several companies have developed instrument based POC devices (consisting of a platform and cartridges) for HIV monitoring, to count CD4 outside a laboratory and most recently to test for viral load at or near POC, some of them using the Xpert platform (Medecins Sans Frontieres, 2017). This technical integration at diagnostic device level is likely to impact programmatic integration of the two disease control efforts as well.

The paper draws on two sets of fieldwork conducted between July 2015 and January 2017 among actors involved in diagnostic development for tuberculosis and HIV, including end-users and those involved in designing, deploying, funding, and evaluating diagnostics. The first set of material consists of 52 semi-structured interviews with diagnostic developers, donors, members of civil society, industry consultants, international organizations, policy makers, regulators, and researchers who are distributed geographically and act globally. These interviews were conducted in person and via Skype or telephone with participants based in Asia, Africa, Europe and North America and combined with visits to workshops, companies, and conferences in Europe and North America. A second set of material is based on fieldwork in Bangalore, India, including 15 interviews with diagnostic developers, decision-makers, NGO program officers, scientists, TB and HIV program officers, laboratory managers, technicians and nurses using TB and HIV diagnostics as well as visits to companies, clinics, and laboratories. The study was approved by UMREC, the ethical review board of Maastricht University.

The topic list guiding the interviews covered establishing the participant's involvement with diagnostic development, views on the diagnostic technology in question, steps of development including the actors involved; handling of conflicts, failures or challenges; evaluation and regulatory practices; difference of POC diagnostics to lab-based tests, understanding of the point of care; and practices of making the diagnostic work at point of care. Analysis and data collection emerged iteratively. The fieldwork notes, interview notes and transcripts, for those that were electronically recorded (all but five), were analyzed using Nvivo. The coding scheme was developed based on the topic list, research question, notes and fieldwork reports, and codes that emerged from reading the material.

## Findings

### Target Product Profiles and Engineering Advice: Dominating How Simplicity Is Valued

In the global health literature, journal editorials, and policy documents, much of the hope around POC testing as a mean to cut diagnostic delay centers around the idea of simplicity. As stated earlier, such simple diagnostic devices would allow providers not specialized in laboratory techniques to perform testing in settings with less resources and equipment than a centralized laboratory (Pant Pai et al., 2007; Peeling and Mabey, 2010; Denkinger and Pai, 2012; Toskin et al., 2017). Our results reveal how donor groups such as Bill and Melinda Gates Foundation, USAID, Foundation for Innovative New Diagnostics (FIND) or UNITAID have considerable influence in defining what is good in relation to the register of simplicity; especially in their role as funders of research and development activities of diagnostic developers and through publication of design standards. Dominant definitions of simplicity include the following themes “not more than 3 user steps,” “uncomplicated sample preparation,” “rapidity simplifies access,” and “simplicity within the device.”

#### “No More Than 3 User Steps”

Most developers we interviewed had been advised by their funders to minimize user steps and to automate to keep user errors low. A former programme officer of a global funding agency explained that molecular POC devices, consisting of an instrument and not a disposable test, are considered simple if they are fully automated and involve less than three user steps so as to not depend on personnel, infrastructure, qualifications, or (re)training (Scientist 3). A TB test developer referred to the concept of plug and play, meaning the device runs by itself once turned on, requiring minimal expertise by the operator:

“*I think by removing all aspects that would require trained personnel, was the first point because it is easy to say well you just centrifuge it. And you just transfer 5oo microliters and then you just and then you just…., I think all those things are not real just when you see the facilities that these kinds of clinics have. So I think it's a case of taking all that out and make sure that it should literary be plug and play with zero expertise”* (Test developer 19).

The idea of minimizing user steps to 2–3 is also included in the Target Product Profiles (TPPs) published by the WHO. This document lists the optimum and ideal conditions new TB tests should fulfill for different purposes across a health system. The TPPs document was developed with input from a range of global stakeholders in a Delphi process overseen by FIND, a Geneva based Foundation involved in the development, evaluation, and implementation of diagnostics for poverty-related diseases. The document was finalized at a 2-day consensus meeting in 2014, held by the WHO, attended by representatives from technical agencies, research institutes, supranational reference laboratories, national TB programs, industry and clinicians, implementers, and a patient advocate. The TPPs document explicitly mentions that new TB tests should be as simple to perform as Xpert MTB/RIF, a cartridge-based molecular diagnostic platform, which is described as consisting of 2 steps (World Health Organization, 2014). The TPPs therefore define one step or no step of sample preparation as the optimal criteria and maximum 2 steps as the minimal criteria for a rapid biomarker test (including assay processing steps), a rapid sputum based test for detecting TB at microscopy levels, and a next-generation drug-susceptibility test to be implemented at peripheral levels of the healthcare system. Precise volume control and precise timing is not required. The reasoning is given as follows:

“*The Xpert MTB/RIF assay has 2 steps. A new test should be as simple to perform as Xpert MTB/RIF. Devices such as a centrifuge or heat block are available only infrequently at the level of microscopy centres; therefore, they should not be required for novel assays. The expertise needed to operate a micropipette is also often lacking”* (World Health Organization, 2014, p. 58, 73, 84).

Interestingly, the initial Delphi survey results specified that a rapid sputum based test for detecting TB at microscopy levels could have a maximum of three steps as a minimal criteria if no steps require precise volume control. It also stated that: “*In general, it is not so much the number but the types of steps to be performed*” (World Health Organization, 2014, p. 29). After the consensus meeting, this explanation had been omitted in the final TPPs document and the number of steps had been reduced to two.

The WHO published the TPPs as an instrument to incentivize developers to enter the TB market (similar profiles have since been released for other diseases). They specify design targets and as such guide developers in their efforts. This is particularly welcomed by smaller start-ups who are often unfamiliar with the global health market dynamics and user needs. The TPPs developed into a way of talking about what future TB tests should look like (Scientist 3). What is more, developers who have diagnostics that do not fit the TPPs find it more difficult to gain funding and support by global players such as the Bill and Melinda Gates Foundation or FIND (Test developer 4). By defining global design standards and a language to talk about new diagnostics, a specific way of valuing simplicity is being enacted and comes to dominate.

#### “Uncomplicated Sample Preparation”

A crucial aspect of simplicity for HIV POC testing, as propagated by the Bill and Melinda Gates Foundation, is a finger stick and not venous blood collection method. The latter would involve more complicated pipetting or user steps. For developers, this simplification of user steps complicates the development process. It means the sample volume that a tester draws is low, the sample needs to be translated into plasma, the blood needs to be separated, and the diagnostic needs to be able to detect very little virus copies in a small sample volume (Test developer 11). Dominant ways of valuing simplicity by global intermediaries or funders can also influence development of a diagnostic through direct engineering advice. Currently, patients under investigation for TB spit into small plastic cups that close with a screw-on lid. The healthcare worker needs to reopen these to further process the sputum for testing, in some settings without masks or other safety precautions. A test developer recounted how they were advised to not engineer these sputum cups, for instance, so that there was no need to reopen them, because it would complicate existing protocols and logistics of public TB programs (Test developer 5). The register of uncomplicated sample preparation clashes with the value register of “biosafety” by health care workers in Malawi. When the developer had consulted them, they had suggested that a good POC diagnostic should offer a biosafe way to handle sputum samples.

#### “Rapidity Simplifies Access”

Short turn-around-time is considered an important part of simplicity too. Several research studies show that delays in making TB diagnostic results available are an important factor in losing patients to follow up (Storla et al., 2008; Claassens et al., 2013; Sreeramareddy et al., 2014). Donors therefore assume that rapid diagnostics fit better into busy clinic workflows and simplify accessibility of these technologies for patients. Results would then be available during the same clinic visit, as opposed to two visits for first providing a sample and then returning for results. According to a WHO officer, such assumptions are often untested and hard to change, yet they are complicated when devices are introduced into clinics (officer WHO 3).

#### “Simplicity Within the Device”

The simplicity should not only be in the user steps but also inside the device. A TB test developer explained that they aim for simplicity also inside “the box” to minimize machine errors and to reduce cost:

“…*And then really just to keep the complexities as low as possible so you reduce the failure rates [of the diagnostic device]. And just by not getting sucked into anything that is never going to be financially feasible, because there are so many great technologies out there and it is so tempting to think ‘well that one is great let us go with it', have a constant appreciation of the cost”* (Test developer 19).

A senior officer at FIND confirmed that one of the criteria by which they scout and select new technologies that they support, is that it should not be technically too complicated, e.g., involve too many steps within the device. However, the FIND officer also cautions that this might not be possible. Diagnosing TB involves liquefying sputum, amplifying DNA and detecting TB which requires a lot of fluid moved around inside a device and technology to do so (Officer FIND 1). Each of those steps, when built into a device or onto a cartridge, can cause complications, potential errors, require intellectual property rights or increase the cost of the diagnostic platform and individual test. This example shows that how the dominant actor values simplicity can change according to when and where this actor wishes to apply the register. It also shows the strong association of valuing simplicity with cost-effectiveness.

### Redefining What Is Simple Based on Different User Understanding and Why Automation Is Useful

While many of the developers adopt these dominant ways of valuing simplicity, others have slightly different understandings of simple diagnostics and of the reasons for why they should be simple.

#### “Quality of User Steps, Not Number”

A test developer took issue with defining beforehand the number of user steps because it assumes users in low resource settings cannot be trusted to do more than three steps. According to him, it is not so much the number but the quality of these steps. A laboratory technician diagnosing TB in a public microscopy center does not have much career prospect and therefore values learning on the job and being respected for doing the job right (Test developer 21). The developer arrived at a different definition of simplicity than the global donor community. Automation and reduction of user steps is good for certain reasons. For example to allow walk away time to do other things in busy laboratories or to standardize an unregulated private sector (Test developer 21). Many countries struggle with unregulated private sector laboratories and poor quality testing. As a result of the automation within the device, an Indian public health TB programme officer accepts test results by Xpert MTB/RIF from the private sector, without asking patients to retest in the public sector first before initiating free multi-drug resistant TB treatment (Implementer 3).

Similarly, test developers and implementers with several years of experience in innovating HIV diagnostics for the POC argued that simplifying should be done with a good understanding of the user situation (Test developer 5, 11). Users at POC mainly focus on patients and not the operation and maintenance of diagnostic devices. Aside from reducing the number of steps and simplifying sample collection, automation should also not require precise timing (Test developer 11). The benefit of automation, and therefore these actors' register of simplicity, is ultimately depending on the kind of user, the site and the quality of those steps that need to be undertaken.

#### “Simplify Transportation”

Others challenge the idea of simplifying instruments and instead attach their register of simplicity to other diagnostic processes where simplification should occur. A programme officer at Medecins Sans Frontieres argued that any kind of instrumentation involved beyond a glucometer test is unsustainable at POC, because of the quality assurance and maintenance requirements which pose unsustainable burdens on existing health systems and staff. Instead, transportation to central laboratories could be simplified by not requiring samples to arrive timely or in certain storage conditions, for instance through dried blood spot technology for HIV related testing. This interviewee doubted whether molecular testing at POC is always the best solution and pointed to activism and market interests pushing for certain solutions (Officer Medecins Sans Frontieres 2). Similarly, a scientist at an international technical agency argued that Xpert simplified important but not all aspects of the testing process: “*…if you look at the whole infrastructure, the lab got reduced to a coffee machine on the table (…) [and] the test got reduced to a cartridge*” (Scientist 3). But Xpert did not simplify sputum collection and transport, logistics or information flow and sample type (Scientist 3). It reveals the choices that were made in what is considered simple.

The examples provided above show that what is valued as simple differs between actors and refers to different things: the biomedical platform, the workflow/user steps, the transportation or the sample collection. At the same time, the act of defining a technology as simple adds value and is valorizing. A test developer's notions of simplicity evolve over time and are shaped by global intermediaries and changes in market. TPPs and imaginaries of users, as propagated by powerful global health actors, play important roles in how simplification is negotiated and shape how diagnostics are being developed. Many of the developers agreed with the dominant way of valuing simplicity, that automation and lesser human interface is better, assuming it reduces chances of error and thus increases accuracy. This view acknowledges that technically—inside the box—diagnostics can be complex as long as they are simple for the user. Yet, the emphasis on simplicity within the device—to keep cost of maintenance and the device low—also reveals how simplicity could stand for cost-effectiveness *per se*. If the diagnostic is simple and can be operated in given constraints of equipment, human resources and workload, then it does not require additional costs of changing or ameliorating these constraints. Hence, investing in better infrastructure, more staff or strengthening of the health and laboratory system are not an issue. Other developers and program officers proposed alternative definitions of simplicity and reveal different user understandings (dealing with patients not only tests) and different reasons why automation might be helpful, depending on a combination of type of users, existing, and competing diagnostic practices (unstandardized private sector), site and quality of steps to perform. They adapt, modify, reject, or compromise the dominant way of valuing simplicity.

### Implementation Practices, Complicating Simplicity, and Prioritizing Other Value Registers

The process of valuing applies not only to design and development practices but also to how/which technologies are put into use. During implementation, healthcare workers, patients, and decision makers might prioritize workflow, patient pathways, integration with other ongoing testing and costs over simplicity. This complicates simplicity and has important implications for utilization and access of these diagnostics, as described below.

In a small public clinic in Bangalore, the lab technicians of an integrated counseling and testing center (ICTC), run by the National AIDS Control Organization, collected the blood from patients needed for HIV rapid testing through venous blood rather than a finger prick, which enabled them to draw more blood for follow-up tests (Laboratory technician 3). This challenges the idea of “uncomplicated sample preparation” and is contrary to the device developers' expectations.

Patients might perceive a diagnostic to be too simple or mundane for its price if compared to other, more revealing, diagnostic services that can be purchased for a similar price. In the Indian private sector the high cost of the Xpert MTB/RIF (even at the government subsidized price) means it competes with other seemingly more revealing and in this sense less simple diagnostic technologies. A CT scan analyses the entire body whereas the Xpert only tests sputum. As a result, doctors do not offer Xpert MTB/RIF to avoid losing clients who might take offense at being handed a simple, yet expensive, test:

“*the person who is practicing in a neighborhood, (…) the doctor says, if I write the [Xpert] test, patient may not come to me next time, okay, like it's costing two thousand rupees, just imagine CT scan is less than that! (…) a CT scan is considered as advanced, more advanced test, you just test the sputum and charge two thousand rupees in neighborhood clinics and hospitals? Definitely that guy will get a backlash from the patient”* (Laboratory manager 4).

In many clinics, the way HIV rapid tests, CD4 monitoring, and Xpert MTB/RIF tests are embedded and used within diagnostic practices increases their actual processing time from 20 or 90 min to 1–2 days. This has to do with the way patients are registered, samples are processed and batched before running the device, and the way reports are authorized and communicated to patients. In the HIV testing center mentioned above, for instance, the laboratory technician batched HIV testing twice a day instead of running the test immediately when the sample is taken. This increased patient waiting times. It also meant this laboratory technician preferred a different test design, namely bulk packaging of test strips that would allow him to open eight tests at a time instead of the single-wrapped test strips the developer provides. Opening single strips takes much more time, especially when batching 30 tests (Laboratory technician 3).

During implementation, simplicity is interacting with workflow and waiting time, which has crucial implications for the design of diagnostics and stability requirements for reagents. Instead of one longer visit, patients might value two short visits; instead of short turn-around times and minimum preparatory steps, healthcare workers might care more about how long a sample occupies the device. A WHO officer illustrated this with the example of a HIV monitoring test:

“*Those people who were not used to same day results, they were used to going, giving blood and coming back the next day. It would take them two visits but those visits were very short. Whereas with the introduction of point of care testing, with small instruments that have a fairly low throughput in maybe 2 specimens an hour max, (…) they are waiting all day long just to see if they can get their specimens onto the instrument. And what we saw was happening was they [staff] were taking the sample, putting it into the cartridge and then just sitting on the bench and waiting till the instrument was free. And those cassettes are not designed like that. (…) We said [to manufacturers] you should consider what needs to be the stability of the specimen once it's in these cartridges. Because the second you can close the cartridge it is easy to just put it down and not put it in the machine”* (Officer WHO 3).

Some diagnostic devices for HIV monitoring require incubation and sample preparatory steps for 15 min but the time in the device is only 2 min. The more automated ones have these steps integrated which means the device is in use for 20 min. At times stability is more important than how fast a healthcare worker can read a result: The faint line of a HIV rapid test kit proved too difficult to read in a busy public clinic in Bangalore. The result was not stable beyond 20 min and healthcare workers were not always able to read results within this specified waiting time. This produced wrong results and was not useful, especially because confirmatory testing was not available at this clinic (Implementer 2). Because the test did not fit into the busy workflow at the clinic, it was relocated to a more centralized laboratory with more attention by dedicated staff to adhere to waiting times, and with other diagnostics that could contradict or support the test result. Moving POC diagnostics to more centralized locations means they are less accessible for patients who then have to invest more time, money, and effort to reach them. It also shows that diagnosing might involve many more steps for patients even if it only takes three user steps for health care workers. Hence, one might ask, who is simplicity for?

### Valuing Accuracy in Global Evidence Making Practices: Accuracy Trumps Accessibility

These above examples show how different value registers of workflow, patient pathways and cost are competing with the dominant way of valuing simplicity. These competing values are not recognized in the global evidence making practices on new diagnostics. Here, primarily, the register of accuracy or technical quality is tested. Agencies such as the WHO apply a technical evaluation process for regulatory approval and guideline decision-making processes, developed for laboratory-based technologies before POC testing existed. Sensitivity and specificity of a new test are compared with those of the laboratory standard (Palamountain et al., 2012). To be considered distinguishing disease from health accurately, a diagnostic needs a certain degree of sensitivity (how often it provides a positive result in people who really have the disease) and specificity (how often it provides a negative result in people who do not have the disease). Aspects of simplicity, workflow or patient pathways are not consistently assessed or measured. Reaching higher sensitivity/specificity is often linked with cost, of either the materials and techniques used inside the devices or the equipment and infrastructure required to maintain or conduct the diagnostic. In the case of the Xpert MTB/RIF, reaching a higher accuracy meant a longer development time. The developer justified this with potential profits: a test with higher accuracy can also be sold in high-income markets (Test developer 18). Yet, what precisely is valued as a high (enough) sensitivity and specificity for the purpose at hand depends on an actor's value register and how the register of accuracy interacts with other values of cost and access.

The WHO assesses the sensitivity and specificity of new diagnostics based on independent evaluation studies. For TB detection, the Xpert MTB/RIF has become one of the reference or gold standards. New TB diagnostic need to be at least non-inferior in performance compared to the Xpert MTB/RIF with a sensitivity of 89% and a specificity of 99% (Steingart et al., 2014), unless they are designed for an entirely different setting or purpose. The Xpert not only defined a gold standard it also set a market price at 10USD per test. This is what donors are willing to spend. Yet, the 10USD price point was facilitated by global donors through a buy down. Several of the test developers we interviewed doubt that a similar buy down would happen again for other diagnostics. The high accuracy in combination with a 10USD price point has set a very high bar for competitors who might have to compromise accuracy in order to get cost down and achieve test volumes (Test developer 20).

Several test developers we spoke to criticized the fact that POC tests are expected to be as accurate as laboratory tests (Test developer 8, 20). A test developer and a TB scientist argue that the global community could give precedence over aspects addressing accessibility of diagnostics. This could mean adjusting or giving up to some extent the requirements for accuracy and to reflect that type of flexibility in the TPPs (Scientist 3, test developer 20). To underline this argument, a test developer refers to rapid HIV tests that do not live up to the same sensitivity standards as laboratory-based tests, but trump in terms of accessibility:

“*So when the rapid diagnostic test came out for malaria, or for HIV, were they as sensitive as the lab Elisa? Far from it. (…) They got maybe 50% of the sensitivity. But that is one innovation that has had tremendous impact on these diseases (…) because they were available where they needed to be available. (…) So when you are talking about point of care test, you can't in my opinion have the same benchmarks as gold standard laboratory tests”* (Test developer 20).

Accuracy is often competing against accessibility and cost. Should concerns over accessibility trump those over technical quality? In a priority setting exercise by way of an online survey among 33 country stakeholders of 14 countries, participants ranked the elements of the TPPs document in order of importance. Sensitivity was ranked highest (Adepoyibi et al., 2018). This exercise was overly generalized, not representative of varied users and not adjusted to specific situations in which, as suggested by our interviewees, users would prioritize different aspects of TPPs to make diagnostics work. It shows that it is hard to go back on one value register, alter the hierarchy of functions attributed to a diagnostic and introduce a test with lower technical accuracy for a similar purpose (for instance by making it less accurate even if it allows optimizing accessibility). But how to define a well-accessed test? Equal access is yet another value register with different ways of enacting it. The Xpert MTB/RIF is widely underutilized due to cost and operational constraints and only available to limited patient groups in more centralized locations. This creates differential access for users. But the Xpert also offers a better diagnosis for those who can access the diagnostic, and benefit from the changed epidemiological definitions of multi-drug resistant TB, and the related interest and funding. A WHO officer wondered where diagnostics like the Xpert should be deployed: to busy sites reaching a maximum number of people or to those sites that few people access but who otherwise would not be diagnosed at all (WHO officer 3). Her reflections show that this is also a choice about building infrastructure, investing in specimen transport and electricity, and developing new business models. Busy clinics might not need POC devices but could be served by improved sample transport to laboratories, while POC devices could be deployed at sites with very low number of patients tested but where public health impact is important in terms of providing access to everybody (WHO officer 3).

To sum up, the tensions between different valuation practices, accuracy, cost and access and the way they are enacted, directly impact design of diagnostic algorithms and technologies, investment decisions and regulation, and ultimately the societal impact and accessibility of a diagnostic. In the dominant way of valuing accuracy, tests need to be as accurate as existing tests with a similar purpose. Competitors and global evidence-making practices shape the dominant value register of accuracy and define the market, funding for research, and set standards against which developers are assessed in a very literal way during evaluation studies. Valuation practices change over time, for instance when new types of diagnostic devices emerge. The Xpert changed what new POC diagnostics for TB need to achieve at POC to be considered “good” in evaluation studies and the global policy arena.

## Discussion

In this paper, we identified multiple values that are at play when innovating POC diagnostics. Depending on the moment, specific values come to the fore (simplicity in design stages, accuracy in evaluation, and workflow during use), pointing to different values (scientific, economic, public health, political, regulatory, personal health) that are being generated through diagnostics for different actors. Heuts and Mol argue against schematizing the insights of valuing because it is impossible to draw conclusions about what is good across different cases but also within a single case, such as the tomato. The actors who are valuing tomatoes do not seem to miss a theory of what is a good tomato in their valuing practices (Heuts and Mol, 2013). Contrary to the tomato case, in the case of POC diagnostics there is a striving for control and standards among global intermediaries and the WHO. Absence of a theory of what constitutes a good POC diagnostic for TB control was identified as one of the reasons impeding development of new diagnostics (Pai, 2013). Global health diagnostics that are simple to operate and maintain, and do not require sophisticated laboratory environments and user skills, seems a clear selling point and a guiding principle to assess what is a good POC diagnostic. Simplicity is believed to add to the stickiness of these technologies, to avoid too fluid diagnostics that would produce unreliable results and to ease their travel to peripheral testing sites across different countries and settings. We tried to show how in the dominant way of valuing simplicity, there is also a strong association with cost-effectiveness pointing to a possible conflation of value and price. Contrary to a laboratory based diagnostic, for POC diagnostics, simplicity is part of what makes a good POC test. It helps if you can label a diagnostic as simple, it is performative in the sense that it attracts funders and aligns with global design standards, it legitimizes and adds value—simplicity is valorizing. Yet, simplicity is not enough value, it interacts and competes with other valuation registers of accuracy, cost, workflow, or patient pathways.

However, when it comes to global evidence-making practices and developing guidelines on new diagnostics, these do not reflect the above mentioned tensions and instead prioritize accuracy. Global health is ripe with assumptions of direct causal reactions often linked to technological innovations that do not reflect the complicated reality of improving clinical care and health systems. The drive toward POC testing can be seen as part of a larger shift in what counts as an intervention in the global responses to infectious diseases: away from preventive approaches addressing behavioral and broader social changes toward targeted technical and measurable solutions (Adams, 2016; Mahajan, 2018). What is more, global health interventions often have specific ambitions of scaling up in time and space. In a similar way it is assumed that POC diagnostics will be rolled out countrywide and within a limited time period. Yet, those ambitions are not systematically tested in the global evidence making practices. Our results highlight alternative ways of valuing simplicity that challenge some of these temporal assumptions related to scaling. Hence, the need for building different types of testing infrastructures, paying more attention to situation specific adaptations and broadening the criteria of evaluation. Regulatory authorities and procedures to evaluate new tests, which were established before POC diagnostics were available, should include these operational and contextual aspects (Palamountain et al., 2012). Our analysis shows that this requires critical reflections on three key questions. First, where is simplicity in these value registers located? Is it inside the device, in the user steps or in other parts of the diagnostic process? Next, who are the users for whom these diagnostics need to be simplified? Lastly, what is the purpose of these innovations? Do these aim at widening access and providing greater equity of care or keeping costs of the device low and/or avoiding investments in infrastructure for delivering equitable healthcare?

Our analysis shows how valuing simplicity has direct effects on how diagnostics are aligned to settings of intended use and ultimately on the utilization, equity and accessibility of a diagnostic. The politics of framing and developing new diagnostics, dominant interests and values of donor groups and global intermediaries supporting developers, overrule alternative or competing registers of the healthcare users. Further, ethnographic research of diagnostics in use shows how actors perceive and enact the added value of HIV or malaria rapid tests or the Xpert MTB/RIF to diagnose TB differently within different contexts and settings (Angotti, 2012; Hutchinson et al., 2014; Engel et al., 2017a). In the case of the Xpert MTB/RIF for diagnosing TB in children, for instance, Indian healthcare providers use it as a first diagnostic TB test, as an initial screening test to rule out TB as well as a confirmatory test after TB diagnosis is established or for drug susceptibility testing. This has an important positive impact on the speed of diagnosis, improving treatment for and awareness of drug resistance in children (McDowell et al., 2018). Importantly, in doing so, they are not only redefining the norms and value frames imposed by the developers and international funders but also redrawing the boundaries between the normal and the pathological.

While this literature has for the most part focused on end-users of diagnostics, our study juxtaposes different viewpoints and valuation practices from actors across the design, development, and implementation spectrum. The case of developing POC diagnostics, therefore, also shows how the evidence underpinning these new technologies is framed from the top down and adapted or even ignored through the practices of end-users in their local therapeutic milieu. When making these therapeutic tools and diagnostics work in local practices, dominant understandings of simplicity are complicated and dominant, global valuation practices are also being challenged. Yet, in these top-down/bottom-up dynamics, local user practices cannot un-do entirely the design choices and selections that have been made earlier. Just like in the tomato case, where the competition between different actors' registers might overrule the consumer's values and lead to a tomato in the supermarket that is not particularly tasty, powerful global health actors, and donors dominate these valuation processes. They determine which innovations are being supported and how they are being developed and evaluated by defining design targets, funding mechanisms and setting market and evaluation standards.

Finally and more importantly for this research topic, one of the consequences of the focus on seemingly simple technologies as solutions to complex problems of access is underfunding of health infrastructure, capacity and training resources for addressing some of the existing structural inequalities. Consequently, this means that the way dominant actors currently value POC diagnostics risks exacerbating health inequalities between those who do and do not have the necessary social and financial resources to access better equipped and functioning healthcare facilities.

## Data Availability Statement

The datasets generated for this study will not be made publicly available. Public deposition of the data would compromise interviewee privacy. Data are available upon individual request with the necessary editing to preserve anonymity of the study participants.

## Ethics Statement

The studies involving human participants were reviewed and approved by UMREC, the ethical review board of Maastricht University. The patients/participants provided their written informed consent to participate in this study.

## Author Contributions

NE conceived and designed the study, and collected and analyzed the data. NE and AK wrote the paper.

## Conflict of Interest

The authors declare that the research was conducted in the absence of any commercial or financial relationships that could be construed as a potential conflict of interest.
